# Sperm DNA damage and assisted reproductive technologies: reasons to be cautious!

**DOI:** 10.1186/s12610-016-0038-3

**Published:** 2016-09-27

**Authors:** Joël R. Drevet

**Affiliations:** GReD Laboratory, CNRS UMR6293 – INSERM U1103 – Clermont Université, Clermont-Ferrand, France

## Is there a more important situation where one should apply the precautionary principle than in assisted reproductive technologies (ART)?

Although ART are formidable answers to the distress of couples having difficulties to conceive, these technologies are far from being totally well controlled. The worldwide increasing demand on ART is by itself an alarming situation that should promote more research on the multifactorial origins of decreasing human fertility. However, the clinical success of ART (although perfectible!) has essentially produced an opposite effect with a slow-down in the effort and money dedicated to the understanding of human infertility. Why bother to understand what is going wrong when one can bypass most of the blockages observed either structural or functional? While a lot of energy and money have been spent to improve ART success rate, the improvement obtained is not up to our expectations and ART success rate has remained at a mere 25 % overall for the past 30 years. This situation is worth analyzing since it could reveal that either we are looking in the wrong directions to improve ART or that the processes of ART generates stresses which annihilate the expected improvements. At the same time, progress made in basic sciences indicates possible reasons for caution that should be seriously considered by clinicians and geneticists.

### ART and male infertility

ART cover a vast array of technologies aimed at bypassing natural blocks that prevent reproduction when all the necessary basic conditions are not optimally fulfilled. ART cover both male and female reproductive defects that more or less equally share the responsibility for reproductive failures. These reproductive defects can be broadly classified as follows: defective gamete production, defective gamete functions, defective female receptivity for embryo development and abnormal embryo development. ART partially cover the two first causes especially when the origin of infertility lies within the male gametes. This is particularly obvious for the infertile situations were spermatozoa are: not sufficiently numerous (oligozoospermia), not sufficiently motile (asthenozoospermia) or/and structurally abnormal impairing their functional properties (teratozoospermia). It should be noted that in most infertile situations, a combination of these defects is often seen leading to a broad OAT classification of sperm samples (standing for: Oligo-Astheno-Terato-zoospermia). ART respond also quite well to infertile situations where the female genital tract makes it too difficult for male gametes to reach the precise site up the uterine horn where fertilization should take place. This may be due to various female driven reasons that have nothing to do with the spermatozoa fertilizing ability, including for example, an uncontrolled immune response towards the immune stranger sperm cells or a mechanical blockade of the female tubes.

There are several technologies that have been progressively designed to respond to these various infertile situations. They start with intra-uterine insemination (IUI) also commonly called artificial insemination. IUI is often employed to treat situations of low sperm volume, low sperm concentration or weakly motile spermatozoa. IUI is also appropriate to treat unsuccessful conception due to female uterine cervical issues or immunological reactions towards spermatozoa. IUI allows a greater number of spermatozoa to be deposited near the oocyte target thus increasing the probability of fertilization. Spermatozoa are most often obtained *via* masturbation prior to the procedure and generally the female partner has been synchronized by hormonal treatment in order to maximize fecundity. This is the least invasive technology since spermatozoa use natural uterine passages to reach the oocyte. The main concerns associated with such technology are the consequence of ovarian hormonal hyper-stimulation and the increased risk of multiple pregnancies. However, there is a male aspect that is largely ignored and has never been explored. It concerns the possible consequences for spermatozoa exposed to collecting devices (most often made of plastic or glass), various media of defined or not completely defined composition, ambient air and light? This is a concern since in natural conception with mammals reproducing *via* internal fertilization spermatozoa are never exposed to such conditions.

When spermatozoa and oocytes are unable to meet naturally, in vitro fertilization (IVF) is used. As stated in the name, spermatozoa-oocyte interaction here occurs outside the female organs. IVF is classically proposed when female tubes are damaged often as a result of infectious episodes (salpingitis) or extra-uterine pregnancy. IVF is also a solution when the sperm quality is not optimal, especially with poorly motile sperm and sub-optimal sperm counts. Classically, the IVF protocol uses hormonal ovarian stimulation to retrieve simultaneously more than one oocyte in order to increase the chances of successful fertilization. This is necessary because fertilization may not concern every oocyte and all fertilized oocytes will not necessarily undergo the developmental program. Oocytes and spermatozoa are placed together in a plastic device for about one day. Successfully fertilized oocytes start to divide and between day 2-day 3 (4 to 8-cells stage) and day 5-day 6 post-fertilization, as they reach the blastocyst stage, one or more embryos is/are transferred into the uterine horn of the recipient mother. The age of the embryo upon transfer, the number of embryos transferred, as well as the use of luteal hormones to prepare the recipient mother uterus for implantation, rely on decisions of the clinical staff and state-enforced regulations. As is the case with IUI, the major concern with IVF is the consequence of ovarian hormonal stimulation and the risk of multiple pregnancies especially when more than one embryo are transferred. As was the case with IUI, since spermatozoa are mostly obtained *via* masturbation on the day of oocyte retrieval, the issues of non-physiological exposures of the male gametes to the local environment (plastic collecting tube, various media, ambient air and light) exist.

A more invasive protocol which consists in the micro-injection of a single sperm cell into an oocyte has been developed and is known as ICSI for Intra-Cytoplasmic Sperm Injection. It is a sophistication of the IVF procedure where fertilization is also achieved outside the natural compartment. This protocol is proposed very often when sperm counts are dramatically low, and with totally immotile spermatozoa. In the absence of sperm in an ejaculate most often due to obstruction in the male accessory tubules, this protocol may be carried out with spermatozoa retrieved either from the epididymis (MESA: Microsurgical Epididymal Sperm Aspiration) or the testis (TESA: TEsticular Sperm Aspiration) after surgical sperm collection. Because it bypasses all natural barriers preventing the encounter of a “bad” spermatozoon with an oocyte, this technology should be used very cautiously and in particular, requires a very thorough selection of the microinjected gametes (both male and female). Initially designed to be a supplementary alternative intended to be used after unsuccessful IUI and/or conventional IVF, it is now the most frequent protocol used in ART accounting for approximately 60 % of the ART procedures worldwide. Somehow ICSI is a victim of its success, since it answers most of the infertile clinical situations making it the protocol of choice of ART centers around the world. In our world where “time is money” it makes it possible to avoid the lengthy burden of sequentially going through IUI, then standard IVF and only when all these attempts have failed to ICSI. In addition, the success of ICSI comes from the fact that it is quite easy to master since it requires neither elaborate skills nor expensive equipment. Infertility clinics around the world are therefore tempted to use it as a protocol of choice. Although ICSI has been available for more than 20–25 years, and has been optimized in many ways, the take home baby rate has not been improved along the years and it stagnates at a mere 20–25 % [1] (https://www.eshre.eu/Guidelines-and-Legal/ART-fact-sheet.aspx). This may be due to multiple factors. On the one hand, one can explain this absence of improvement because ICSI is increasingly used to answer serious infertile clinical situations for which there was no answer before. On the other hand, it is also possible that the ICSI protocol itself, despite the efforts made to increase gamete selection, generates a great stress on the gametes reducing the chances of success.

### Routine spermatozoa evaluation is behind what would be necessary!

Although there is a wide consensus on the fact that ART success is largely dependent on gamete quality, the criteria that are commonly used to evaluate gamete quality are of poor predictive value. Both gametes should be evaluated, although the two are not equal when it comes to their cell physiology. The female gamete is a metabolically active cell that possesses all the housekeeping systems and molecules involved in its protection and repair, if necessary. On the contrary, the male gamete is a highly differentiated cell, transcriptionally silent, consequently unable to elicit any kind of stress defense response that would normally induce “protective” gene expression. In addition, mature spermatozoa have lost most, if not all, of the protective activities that are usually associated with the cytosolic compartment of any cell. Thus, if exposed to stressors, mature sperm cells may become damaged with no possibility of repair. This may engage them towards necrosis or/and apoptotic-like death preventing their possible candidature for fertilization. Any aggression that will affect spermatozoa membrane functions and mobility will limit the chance of these spermatozoa to fertilize because of the tenuous journey they have to go through in order to reach the fertilization site. *In fine* only a very limited number of “top spermatozoa” will reach the fertilization site and among these highly selected spermatozoa, only one will fertilize an oocyte. Spermatozoa journey to the oocyte is such a difficult path that only the most mobile cells will have a chance to approach their mate. Sperm mobility is directly related to the efficiency of both engine and propeller, the mitochondria in the sperm midpiece and the flagella. It is also directly related to the morphology of the cell and especially that of the head, itself dependent on the state of condensation of the sperm nucleus. This “assault course” that allows only a few top candidates to reach the oocyte may be an evolutionary choice for species depending on internal fertilization, potentially explaining why sperm quality control appears low and sperm heterogeneity is high in most mammals, and especially in humans. Since only the very few most mobile, well built (having a well condensed nucleus which is necessary to give least likelihood of suffering DNA damage) spermatozoa will reach the oocyte, it is hypothesized that evolution has not put pressure on producing a high quality sperm population in these species.

If gamete encounter is the mandatory pre-requisite for fertilization, there is one more issue that governs reproductive success: the integrity of the male and female genetic materials combined at fertilization to create a new individual. The quality of the male and female DNA will control the embryonic program and will partly determine the quality of life of the progeny. Regarding DNA integrity the male and female gametes are not equal. Only the oocyte possesses all the DNA repair activities that take care of DNA alterations wherever they come from. Mature sperm cells are devoid of such DNA repair activities because of their particular cytological differentiation. They are metabolically silent, with virtually no or very low cytosolic activities. They also harbor a highly condensed nucleus that does not allow any DNA repair [2]. Should spermatozoa suffer DNA damage, especially during their post-testicular life, they will carry it with them. If such a DNA-damaged spermatozoa fertilizes an oocyte it will be the task of the oocyte to repair the paternal DNA alterations. This repair of the paternal DNA moiety occurs post-fertilization when the sperm nucleus decondenses into the male pronucleus prior to the first division of segmentation. If the oocyte repair capacity is overwhelmed because of a high level of sperm DNA damage and/or because of a low oocyte repair potential, there is a risk of transmitting to cells of the future individual defective paternal genetic material. In addition, the oocyte repair machinery itself may be at the origin of repair errors that could create *de novo* mutations during the repair process, especially when the level of paternal DNA alteration is high, as there is no cellular process that is 100 % error free. Therefore, it is clear that the male gamete is particularly at stake when it comes to DNA alterations. There are several situations, whether physiological or not, that put sperm cells at risk of DNA damage. Male genital tract infections, local or systemic inflammatory conditions, exposure to chemical stressors, exposure to physical stressors, failing intrinsic protective activities upon aging are examples of situations that may affect the integrity of the sperm DNA material. Sperm handling and processing during the ART procedures also increase the risk of damaging paternal DNA material since they expose sperm cells to situations that are far from being physiological. Media in which spermatozoa are collected, washed, conserved and in some instances selected (Percoll gradients) are examples of non-natural situations where sperm can be exposed to sub-optimal conditions that may have unforeseen deleterious effects. Light exposure of sperm samples when collected, conserved, selected and processed during ART protocols is another example of a situation that has no equivalent when fertilization is natural. When ART is carried out with cryopreserved sperm samples the extent of sperm cell damage due to cryopreservation is yet another example of a non-physiological situation having potentially strong deleterious effects on sperm cells that go beyond the well known impacts of cryopreservation on sperm cell viability and mobility. In all these ART-related situations, the sperm paternal DNA may suffer to such extent that it is possible that the means used to overcome infertile situations may be at the origin of new risks that are to date at most under-evaluated and often even denied.

Based on the main basic causes of infertile situations (as indicated above), it was rather logical to propose that sperm count, sperm morphology and sperm mobility would be gross indicators of male fertilizing ability. However is that sufficient? From my point of view, with the present knowledge we have to date, certainly not. The objective of ART should not be simply taking a baby home. It should go beyond that endpoint and the target should be taking home a healthy baby who will become a healthy child and a healthy adult. Knowing that sperm DNA alterations may challenge these issues and that ART itself may induce sperm DNA alterations [3], particular caution should be taken in using and selecting spermatozoa that show the least possible level of DNA alterations. There are numerous assays available that give a direct or indirect evaluation of sperm nuclear/DNA integrity [4, 5]. It is not the purpose of this letter to expose the specificity, merit or default of each of these assays. The main objective is to emphasize that it is certainly time to complete the routine sperm evaluation of infertile males with additional tests that will give an idea as to the level of sperm DNA/nuclear damage. There is room for new assays that would be more accurate and of a better predictive value. It is true that none of the assays available to date allow the selection of a single top sperm that could be used for ICSI. They only give a global assessment of the level of DNA/nuclear damage in a sperm population, which by itself would be valuable information to have both to predict ART success and to predict genetic risks that may exist following fertilization with such spermatozoa. Such knowledge could also open the way for alternative therapeutic strategies prior to ART that aim at reducing the level of nuclear/DNA damage when possible. For example, since a large portion of sperm DNA alterations are of oxidative nature [6], properly designed antioxidant treatments might prove useful for this purpose [7].
